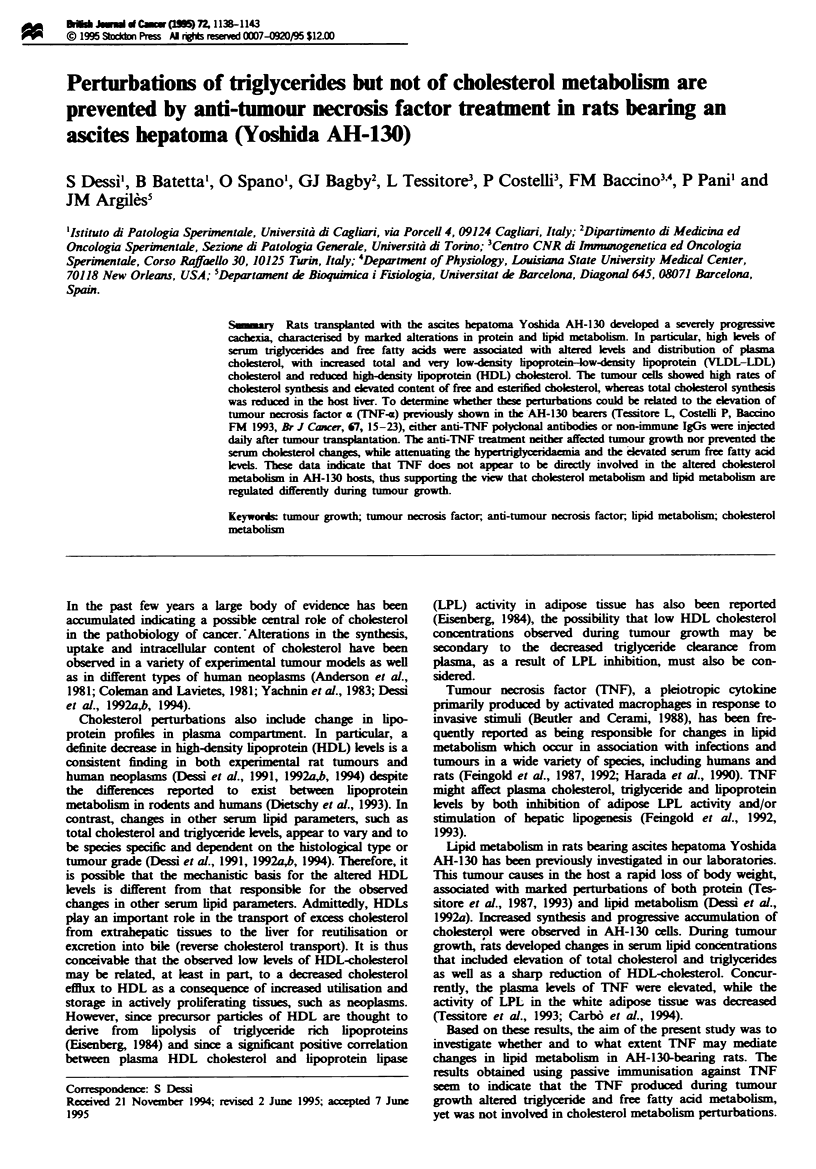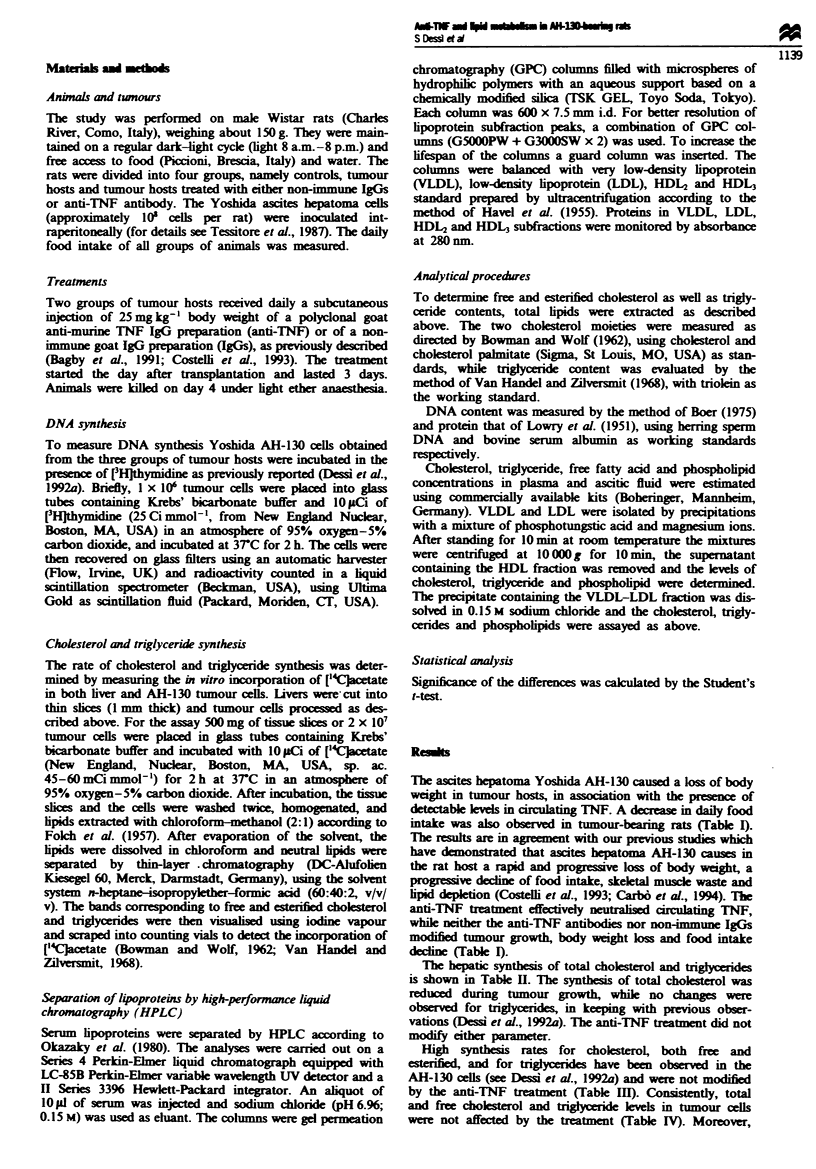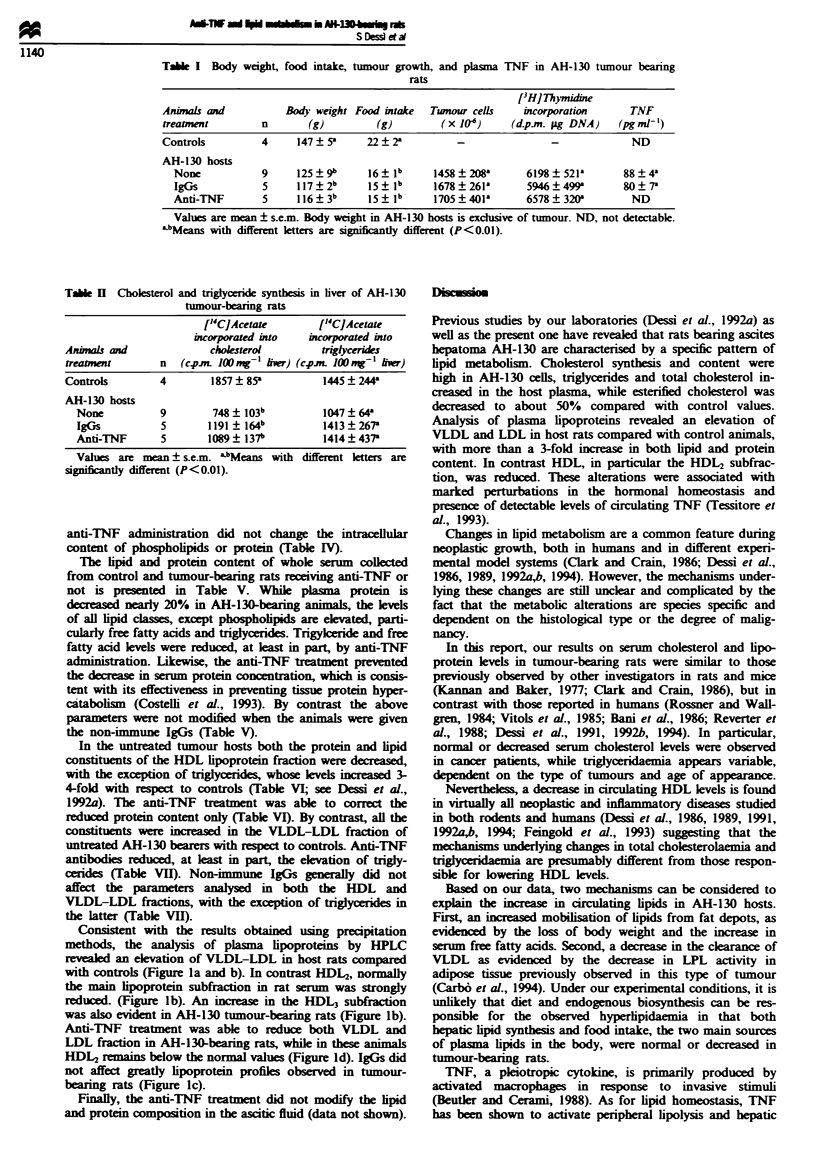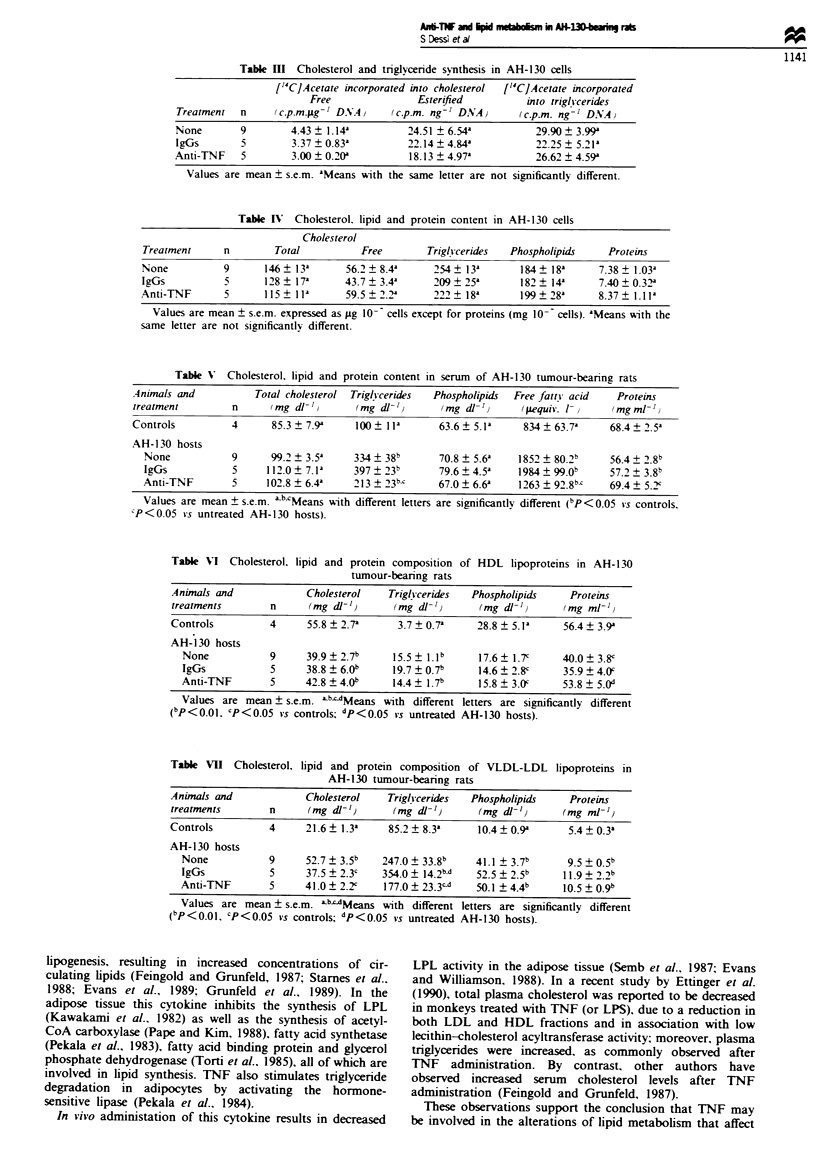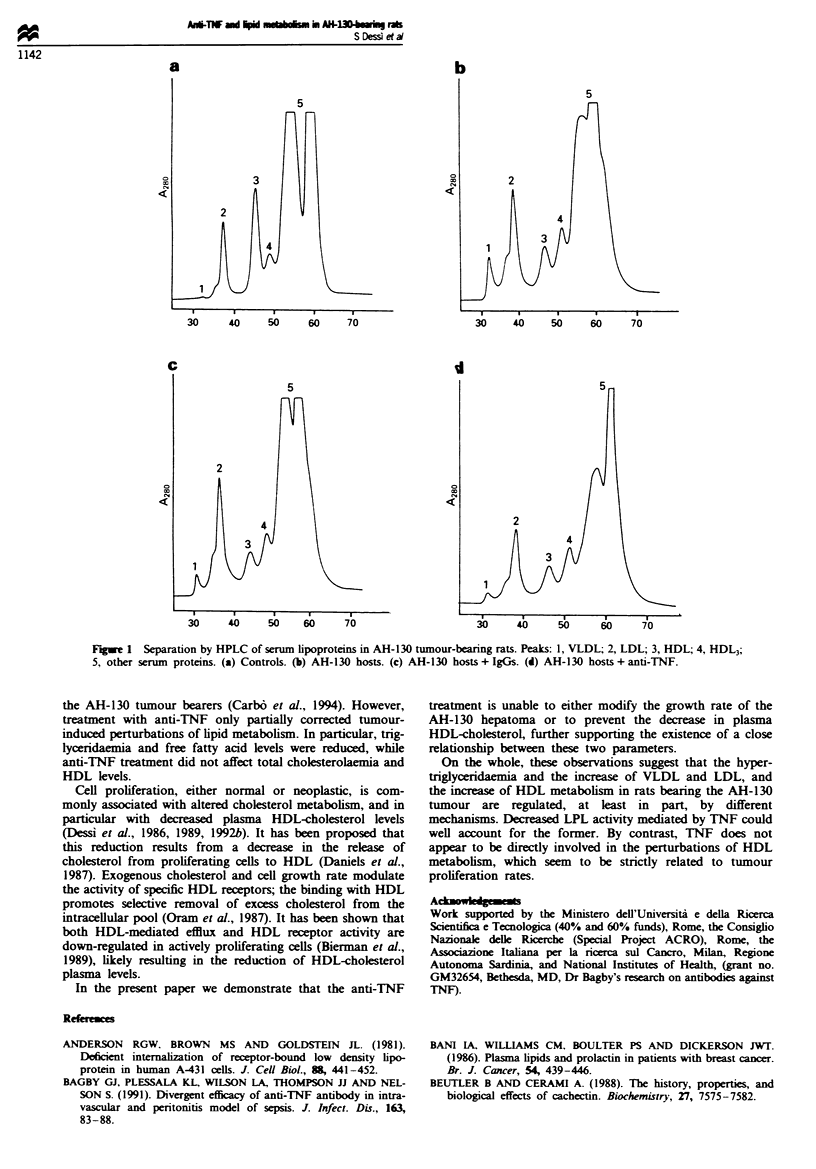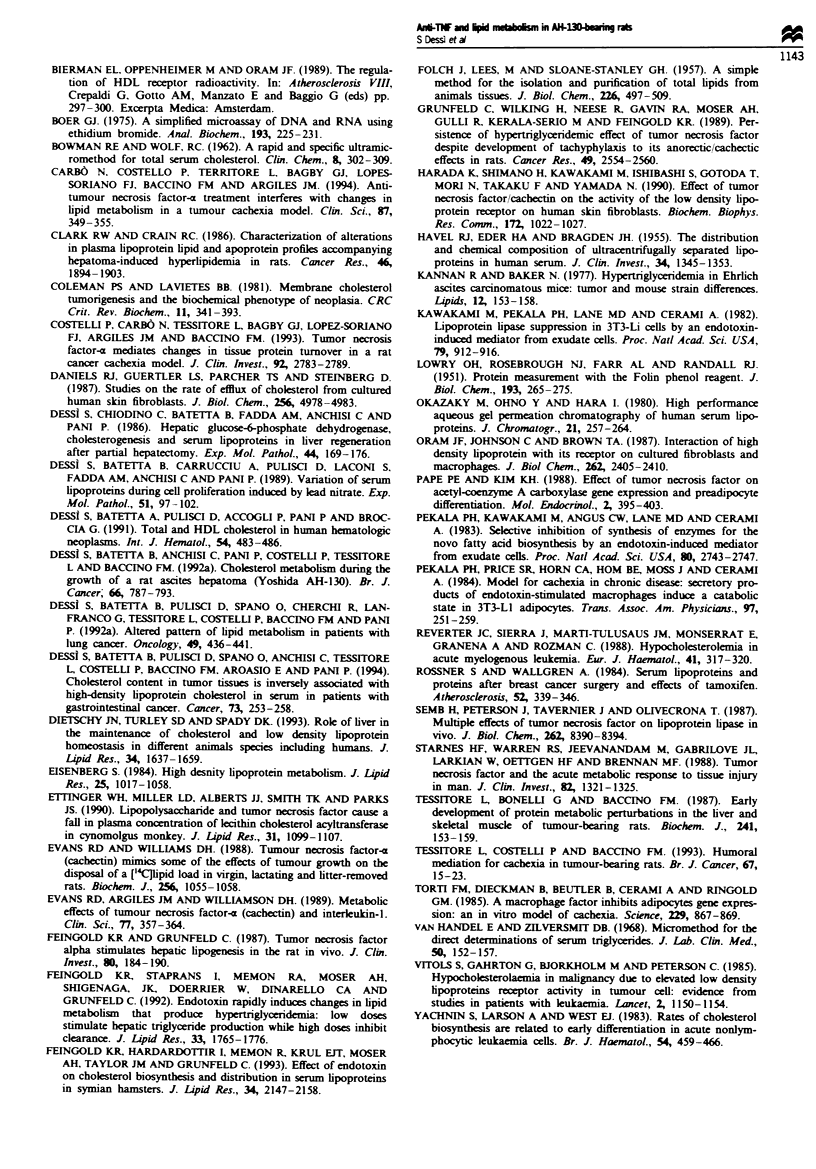# Perturbations of triglycerides but not of cholesterol metabolism are prevented by anti-tumour necrosis factor treatment in rats bearing an ascites hepatoma (Yoshida AH-130).

**DOI:** 10.1038/bjc.1995.477

**Published:** 1995-11

**Authors:** S. Dessì, B. Batetta, O. Spano, G. J. Bagby, L. Tessitore, P. Costelli, F. M. Baccino, P. Pani, J. M. Argilès

**Affiliations:** Istituto di Patologia Sperimentale, Università di Cagliari, Italy.

## Abstract

Rats transplanted with the ascites hepatoma Yoshida AH-130 developed a severely progressive cachexia, characterised by marked alterations in protein and lipid metabolism. In particular, high levels of serum triglycerides and free fatty acids were associated with altered levels and distribution of plasma cholesterol, with increased total and very low-density lipoprotein-low-density lipoprotein (VLDL-LDL) cholesterol and reduced high-density lipoprotein (HDL) cholesterol. The tumour cells showed high rates of cholesterol synthesis and elevated content of free and esterified cholesterol, whereas total cholesterol synthesis was reduced in the host liver. To determine whether these perturbations could be related to the elevation of tumour necrosis factor alpha (TNF-alpha) previously shown in the AH-130 bearers (Tessitore L, Costelli P, Baccino FM 1993, Br J Cancer, 67, 15-23), either anti-TNF polyclonal antibodies or non-immune IgGs were injected daily after tumour transplantation. The anti-TNF treatment neither affected tumour growth nor prevented the serum cholesterol changes, while attenuating the hypertriglyceridaemia and the elevated serum free fatty acid levels. These data indicate that TNF does not appear to be directly involved in the altered cholesterol metabolism in AH-130 hosts, thus supporting the view that cholesterol metabolism and lipid metabolism are regulated differently during tumour growth.


### Perturbations of triglycerides but not of cholesterol metabolism are prevented by anti-tumour necrosis factor treatment in rats bearing an ascites hepatoma (Yoshida AH-130)

S Dessì[1], B Batetta[1], O Spano[1], GJ Bagby[2], L Tessitore[3], P Costelli[3], FM Baccino[3,4], P Pani[1] and JM Argilès[5]

[1]Istituto di Patologia Sperimentale, Università di Cagliari, via Porcell 4, 09124 Cagliari, Italy; [2]Dipartimento di Medicina ed Oncologia Sperimentale, Sezione di Patologia Generale, Università di Torino; [3]Centro CNR di Immunogenetica ed Oncologia Sperimentale, Corso Raffaello 30, 10125 Turin, Italy; [4]Department of Physiology, Louisiana State University Medical Center, 70118 New Orleans, USA; [5]Departament de Bioquimica i Fisiologia, Universitat de Barcelona, Diagonal 645, 08071 Barcelona, Spain.

**Summary** Rats transplanted with the ascites hepatoma Yoshida AH-130 developed a severely progressive cachexia, characterised by marked alterations in protein and lipid metabolism. In particular, high levels of serum triglycerides and free fatty acids were associated with altered levels and distribution of plasma cholesterol, with increased total and very low-density lipoprotein–low-density lipoprotein (VLDL–LDL) cholesterol and reduced high-density lipoprotein (HDL) cholesterol. The tumour cells showed high rates of cholesterol synthesis and elevated content of free and esterified cholesterol, whereas total cholesterol synthesis was reduced in the host liver. To determine whether these perturbations could be related to the elevation of tumour necrosis factor α (TNF-α) previously shown in the AH-130 bearers (Tessitore L, Costelli P, Baccino FM 1993, *Br J Cancer*, 67, 15–23), either anti-TNF polyclonal antibodies or non-immune IgGs were injected daily after tumour transplantation. The anti-TNF treatment neither affected tumour growth nor prevented the serum cholesterol changes, while attenuating the hypertriglyceridaemia and the elevated serum free fatty acid levels. These data indicate that TNF does not appear to be directly involved in the altered cholesterol metabolism in AH-130 hosts, thus supporting the view that cholesterol metabolism and lipid metabolism are regulated differently during tumour growth.

**Keywords:** tumour growth; tumour necrosis factor; anti-tumour necrosis factor; lipid metabolism; cholesterol metabolism

In the past few years a large body of evidence has been accumulated indicating a possible central role of cholesterol in the pathobiology of cancer. Alterations in the synthesis, uptake and intracellular content of cholesterol have been observed in a variety of experimental tumour models as well as in different types of human neoplasms (Anderson *et al.*, 1981; Coleman and Lavietes, 1981; Yachnin *et al.*, 1983; Dessì *et al.*, 1992*a*,*b*, 1994).

Cholesterol perturbations also include change in lipoprotein profiles in plasma compartment. In particular, a definite decrease in high-density lipoprotein (HDL) levels is a consistent finding in both experimental rat tumours and human neoplasms (Dessì *et al.*, 1991, 1992*a*,*b*, 1994) despite the differences reported to exist between lipoprotein metabolism in rodents and humans (Dietschy *et al.*, 1993). In contrast, changes in other serum lipid parameters, such as total cholesterol and triglyceride levels, appear to vary and to be species specific and dependent on the histological type or tumour grade (Dessì *et al.*, 1991, 1992*a*,*b*, 1994). Therefore, it is possible that the mechanistic basis for the altered HDL levels is different from that responsible for the observed changes in other serum lipid parameters. Admittedly, HDLs play an important role in the transport of excess cholesterol from extrahepatic tissues to the liver for reutilisation or excretion into bile (reverse cholesterol transport). It is thus conceivable that the observed low levels of HDL-cholesterol may be related, at least in part, to a decreased cholesterol efflux to HDL as a consequence of increased utilisation and storage in actively proliferating tissues, such as neoplasms. However, since precursor particles of HDL are thought to derive from lipolysis of triglyceride rich lipoproteins (Eisenberg, 1984) and since a significant positive correlation between plasma HDL cholesterol and lipoprotein lipase (LPL) activity in adipose tissue has also been reported (Eisenberg, 1984), the possibility that low HDL cholesterol concentrations observed during tumour growth may be secondary to the decreased triglyceride clearance from plasma, as a result of LPL inhibition, must also be considered.

Tumour necrosis factor (TNF), a pleiotropic cytokine primarily produced by activated macrophages in response to invasive stimuli (Beutler and Cerami, 1988), has been frequently reported as being responsible for changes in lipid metabolism which occur in association with infections and tumours in a wide variety of species, including humans and rats (Feingold *et al.*, 1987, 1992; Harada *et al.*, 1990). TNF might affect plasma cholesterol, triglyceride and lipoprotein levels by both inhibition of adipose LPL activity and/or stimulation of hepatic lipogenesis (Feingold *et al.*, 1992, 1993).

Lipid metabolism in rats bearing ascites hepatoma Yoshida AH-130 has been previously investigated in our laboratories. This tumour causes in the host a rapid loss of body weight, associated with marked perturbations of both protein (Tessitore *et al.*, 1987, 1993) and lipid metabolism (Dessì *et al.*, 1992*a*). Increased synthesis and progressive accumulation of cholesterol were observed in AH-130 cells. During tumour growth, rats developed changes in serum lipid concentrations that included elevation of total cholesterol and triglycerides as well as a sharp reduction of HDL-cholesterol. Concurrently, the plasma levels of TNF were elevated, while the activity of LPL in the white adipose tissue was decreased (Tessitore *et al.*, 1993; Carbò *et al.*, 1994).

Based on these results, the aim of the present study was to investigate whether and to what extent TNF may mediate changes in lipid metabolism in AH-130-bearing rats. The results obtained using passive immunisation against TNF seem to indicate that the TNF produced during tumour growth altered triglyceride and free fatty acid metabolism, yet was not involved in cholesterol metabolism perturbations.

Correspondence: S Dessì
Received 21 November 1994; revised 2 June 1995; accepted 7 June 1995

### Materials and methods

### *Animals and tumours*

The study was performed on male Wistar rats (Charles River, Como, Italy), weighing about 150 g. They were maintained on a regular dark–light cycle (light 8 a.m. – 8 p.m.) and free access to food (Piccioni, Brescia, Italy) and water. The rats were divided into four groups, namely controls, tumour hosts and tumour hosts treated with either non-immune IgGs or anti-TNF antibody. The Yoshida ascites hepatoma cells (approximately $10^8$ cells per rat) were inoculated intraperitoneally (for details see Tessitore *et al.*, 1987). The daily food intake of all groups of animals was measured.

### *Treatments*

Two groups of tumour hosts received daily a subcutaneous injection of $25\ mg\ kg^{-1}$ body weight of a polyclonal goat anti-murine TNF IgG preparation (anti-TNF) or of a non-immune goat IgG preparation (IgGs), as previously described (Bagby *et al.*, 1991; Costelli *et al.*, 1993). The treatment started the day after transplantation and lasted 3 days. Animals were killed on day 4 under light ether anaesthesia.

### *DNA synthesis*

To measure DNA synthesis Yoshida AH-130 cells obtained from the three groups of tumour hosts were incubated in the presence of [$^3$H]thymidine as previously reported (Dessì *et al.*, 1992*a*). Briefly, $1 \times 10^6$ tumour cells were placed into glass tubes containing Krebs' bicarbonate buffer and $10\ \mu Ci$ of [$^3$H]thymidine ($25\ Ci\ mmol^{-1}$, from New England Nuclear, Boston, MA, USA) in an atmosphere of 95% oxygen–5% carbon dioxide, and incubated at 37°C for 2 h. The cells were then recovered on glass filters using an automatic harvester (Flow, Irvine, UK) and radioactivity counted in a liquid scintillation spectrometer (Beckman, USA), using Ultima Gold as scintillation fluid (Packard, Moriden, CT, USA).

### *Cholesterol and triglyceride synthesis*

The rate of cholesterol and triglyceride synthesis was determined by measuring the *in vitro* incorporation of [$^{14}$C]acetate in both liver and AH-130 tumour cells. Livers were cut into thin slices (1 mm thick) and tumour cells processed as described above. For the assay 500 mg of tissue slices or $2 \times 10^7$ tumour cells were placed in glass tubes containing Krebs' bicarbonate buffer and incubated with $10\ \mu Ci$ of [$^{14}$C]acetate (New England, Nuclear, Boston, MA, USA, sp. ac. $45-60\ mCi\ mmol^{-1}$) for 2 h at 37°C in an atmosphere of 95% oxygen–5% carbon dioxide. After incubation, the tissue slices and the cells were washed twice, homogenated, and lipids extracted with chloroform–methanol (2:1) according to Folch *et al.* (1957). After evaporation of the solvent, the lipids were dissolved in chloroform and neutral lipids were separated by thin-layer chromatography (DC-Alufolien Kiesegel 60, Merck, Darmstadt, Germany), using the solvent system *n*-heptane–isopropylether–formic acid (60:40:2, v/v/v). The bands corresponding to free and esterified cholesterol and triglycerides were then visualised using iodine vapour and scraped into counting vials to detect the incorporation of [$^{14}$C]acetate (Bowman and Wolf, 1962; Van Handel and Zilversmit, 1968).

### *Separation of lipoproteins by high-performance liquid chromatography (HPLC)*

Serum lipoproteins were separated by HPLC according to Okazaky *et al.* (1980). The analyses were carried out on a Series 4 Perkin-Elmer liquid chromatograph equipped with LC-85B Perkin-Elmer variable wavelength UV detector and a II Series 3396 Hewlett-Packard integrator. An aliquot of $10\ \mu l$ of serum was injected and sodium chloride (pH 6.96; 0.15 M) was used as eluant. The columns were gel permeation chromatography (GPC) columns filled with microspheres of hydrophilic polymers with an aqueous support based on a chemically modified silica (TSK GEL, Toyo Soda, Tokyo). Each column was $600 \times 7.5$ mm i.d. For better resolution of lipoprotein subfraction peaks, a combination of GPC columns (G5000PW + G3000SW × 2) was used. To increase the lifespan of the columns a guard column was inserted. The columns were balanced with very low-density lipoprotein (VLDL), low-density lipoprotein (LDL), $HDL_2$ and $HDL_3$ standard prepared by ultracentrifugation according to the method of Havel *et al.* (1955). Proteins in VLDL, LDL, $HDL_2$ and $HDL_3$ subfractions were monitored by absorbance at 280 nm.

### *Analytical procedures*

To determine free and esterified cholesterol as well as triglyceride contents, total lipids were extracted as described above. The two cholesterol moieties were measured as directed by Bowman and Wolf (1962), using cholesterol and cholesterol palmitate (Sigma, St Louis, MO, USA) as standards, while triglyceride content was evaluated by the method of Van Handel and Zilversmit (1968), with triolein as the working standard.

DNA content was measured by the method of Boer (1975) and protein that of Lowry *et al.* (1951), using herring sperm DNA and bovine serum albumin as working standards respectively.

Cholesterol, triglyceride, free fatty acid and phospholipid concentrations in plasma and ascitic fluid were estimated using commercially available kits (Boheringer, Mannheim, Germany). VLDL and LDL were isolated by precipitations with a mixture of phosphotungstic acid and magnesium ions. After standing for 10 min at room temperature the mixtures were centrifuged at 10 000 *g* for 10 min, the supernatant containing the HDL fraction was removed and the levels of cholesterol, triglyceride and phospholipid were determined. The precipitate containing the VLDL–LDL fraction was dissolved in 0.15 M sodium chloride and the cholesterol, triglycerides and phospholipids were assayed as above.

### *Statistical analysis*

Significance of the differences was calculated by the Student's *t*-test.

### Results

The ascites hepatoma Yoshida AH-130 caused a loss of body weight in tumour hosts, in association with the presence of detectable levels in circulating TNF. A decrease in daily food intake was also observed in tumour-bearing rats (Table I). The results are in agreement with our previous studies which have demonstrated that ascites hepatoma AH-130 causes in the rat host a rapid and progressive loss of body weight, a progressive decline of food intake, skeletal muscle waste and lipid depletion (Costelli *et al.*, 1993; Carbò *et al.*, 1994). The anti-TNF treatment effectively neutralised circulating TNF, while neither the anti-TNF antibodies nor non-immune IgGs modified tumour growth, body weight loss and food intake decline (Table I).

The hepatic synthesis of total cholesterol and triglycerides is shown in Table II. The synthesis of total cholesterol was reduced during tumour growth, while no changes were observed for triglycerides, in keeping with previous observations (Dessì *et al.*, 1992*a*). The anti-TNF treatment did not modify either parameter.

High synthesis rates for cholesterol, both free and esterified, and for triglycerides have been observed in the AH-130 cells (see Dessì *et al.*, 1992*a*) and were not modified by the anti-TNF treatment (Table III). Consistently, total and free cholesterol and triglyceride levels in tumour cells were not affected by the treatment (Table IV). Moreover,

**Table I** Body weight, food intake, tumour growth, and plasma TNF in AH-130 tumour bearing rats

| *Animals and treatment* | n | *Body weight (g)* | *Food intake (g)* | *Tumour cells (× 10⁶)* | *[³H]Thymidine incorporation (d.p.m. μg DNA)* | *TNF (pg ml⁻¹)* |
|---|---|---|---|---|---|---|
| Controls | 4 | 147 ± 5[a] | 22 ± 2[a] | – | – | ND |
| AH-130 hosts | | | | | | |
| None | 9 | 125 ± 9[b] | 16 ± 1[b] | 1458 ± 208[a] | 6198 ± 521[a] | 88 ± 4[a] |
| IgGs | 5 | 117 ± 2[b] | 15 ± 1[b] | 1678 ± 261[a] | 5946 ± 499[a] | 80 ± 7[a] |
| Anti-TNF | 5 | 116 ± 3[b] | 15 ± 1[b] | 1705 ± 401[a] | 6578 ± 320[a] | ND |

Values are mean ± s.e.m. Body weight in AH-130 hosts is exclusive of tumour. ND, not detectable. [a,b]Means with different letters are significantly different ($P<0.01$).

**Table II** Cholesterol and triglyceride synthesis in liver of AH-130 tumour-bearing rats

| *Animals and treatment* | n | *[¹⁴C]Acetate incorporated into cholesterol (c.p.m. 100 mg⁻¹ liver)* | *[¹⁴C]Acetate incorporated into triglycerides (c.p.m. 100 mg⁻¹ liver)* |
|---|---|---|---|
| Controls | 4 | 1857 ± 85[a] | 1445 ± 244[a] |
| AH-130 hosts | | | |
| None | 9 | 748 ± 103[b] | 1047 ± 64[a] |
| IgGs | 5 | 1191 ± 164[b] | 1413 ± 267[a] |
| Anti-TNF | 5 | 1089 ± 137[b] | 1414 ± 437[a] |

Values are mean ± s.e.m. [a,b]Means with different letters are significantly different ($P<0.01$).

anti-TNF administration did not change the intracellular content of phospholipids or protein (Table IV).

The lipid and protein content of whole serum collected from control and tumour-bearing rats receiving anti-TNF or not is presented in Table V. While plasma protein is decreased nearly 20% in AH-130-bearing animals, the levels of all lipid classes, except phospholipids are elevated, particularly free fatty acids and triglycerides. Trigylceride and free fatty acid levels were reduced, at least in part, by anti-TNF administration. Likewise, the anti-TNF treatment prevented the decrease in serum protein concentration, which is consistent with its effectiveness in preventing tissue protein hypercatabolism (Costelli *et al.*, 1993). By contrast the above parameters were not modified when the animals were given the non-immune IgGs (Table V).

In the untreated tumour hosts both the protein and lipid constituents of the HDL lipoprotein fraction were decreased, with the exception of triglycerides, whose levels increased 3-4-fold with respect to controls (Table VI; see Dessì *et al.*, 1992*a*). The anti-TNF treatment was able to correct the reduced protein content only (Table VI). By contrast, all the constituents were increased in the VLDL–LDL fraction of untreated AH-130 bearers with respect to controls. Anti-TNF antibodies reduced, at least in part, the elevation of triglycerides (Table VII). Non-immune IgGs generally did not affect the parameters analysed in both the HDL and VLDL–LDL fractions, with the exception of triglycerides in the latter (Table VII).

Consistent with the results obtained using precipitation methods, the analysis of plasma lipoproteins by HPLC revealed an elevation of VLDL–LDL in host rats compared with controls (Figure 1a and b). In contrast $HDL_2$, normally the main lipoprotein subfraction in rat serum was strongly reduced. (Figure 1b). An increase in the $HDL_3$ subfraction was also evident in AH-130 tumour-bearing rats (Figure 1b). Anti-TNF treatment was able to reduce both VLDL and LDL fraction in AH-130-bearing rats, while in these animals $HDL_2$ remains below the normal values (Figure 1d). IgGs did not affect greatly lipoprotein profiles observed in tumour-bearing rats (Figure 1c).

Finally, the anti-TNF treatment did not modify the lipid and protein composition in the ascitic fluid (data not shown).

### Discussion

Previous studies by our laboratories (Dessì *et al.*, 1992*a*) as well as the present one have revealed that rats bearing ascites hepatoma AH-130 are characterised by a specific pattern of lipid metabolism. Cholesterol synthesis and content were high in AH-130 cells, triglycerides and total cholesterol increased in the host plasma, while esterified cholesterol was decreased to about 50% compared with control values. Analysis of plasma lipoproteins revealed an elevation of VLDL and LDL in host rats compared with control animals, with more than a 3-fold increase in both lipid and protein content. In contrast HDL, in particular the $HDL_2$ subfraction, was reduced. These alterations were associated with marked perturbations in the hormonal homeostasis and presence of detectable levels of circulating TNF (Tessitore *et al.*, 1993).

Changes in lipid metabolism are a common feature during neoplastic growth, both in humans and in different experimental model systems (Clark and Crain, 1986; Dessì *et al.*, 1986, 1989, 1992*a,b*, 1994). However, the mechanisms underlying these changes are still unclear and complicated by the fact that the metabolic alterations are species specific and dependent on the histological type or the degree of malignancy.

In this report, our results on serum cholesterol and lipoprotein levels in tumour-bearing rats were similar to those previously observed by other investigators in rats and mice (Kannan and Baker, 1977; Clark and Crain, 1986), but in contrast with those reported in humans (Rossner and Wallgren, 1984; Vitols *et al.*, 1985; Bani *et al.*, 1986; Reverter *et al.*, 1988; Dessì *et al.*, 1991, 1992*b*, 1994). In particular, normal or decreased serum cholesterol levels were observed in cancer patients, while triglyceridaemia appears variable, dependent on the type of tumours and age of appearance.

Nevertheless, a decrease in circulating HDL levels is found in virtually all neoplastic and inflammatory diseases studied in both rodents and humans (Dessì *et al.*, 1986, 1989, 1991, 1992*a,b*, 1994; Feingold *et al.*, 1993) suggesting that the mechanisms underlying changes in total cholesterolaemia and triglyceridaemia are presumably different from those responsible for lowering HDL levels.

Based on our data, two mechanisms can be considered to explain the increase in circulating lipids in AH-130 hosts. First, an increased mobilisation of lipids from fat depots, as evidenced by the loss of body weight and the increase in serum free fatty acids. Second, a decrease in the clearance of VLDL as evidenced by the decrease in LPL activity in adipose tissue previously observed in this type of tumour (Carbò *et al.*, 1994). Under our experimental conditions, it is unlikely that diet and endogenous biosynthesis can be responsible for the observed hyperlipidaemia in that both hepatic lipid synthesis and food intake, the two main sources of plasma lipids in the body, were normal or decreased in tumour-bearing rats.

TNF, a pleiotropic cytokine, is primarily produced by activated macrophages in response to invasive stimuli (Beutler and Cerami, 1988). As for lipid homeostasis, TNF has been shown to activate peripheral lipolysis and hepatic

**Table III** Cholesterol and triglyceride synthesis in AH-130 cells

| Treatment | n | [$^{14}$C]Acetate incorporated into cholesterol Free (c.p.m.µg$^{-1}$ DNA) | Esterified (c.p.m. ng$^{-1}$ DNA) | [$^{14}$C]Acetate incorporated into triglycerides (c.p.m. ng$^{-1}$ DNA) |
|---|---|---|---|---|
| None | 9 | 4.43 ± 1.14$^a$ | 24.51 ± 6.54$^a$ | 29.90 ± 3.99$^a$ |
| IgGs | 5 | 3.37 ± 0.83$^a$ | 22.14 ± 4.84$^a$ | 22.25 ± 5.21$^a$ |
| Anti-TNF | 5 | 3.00 ± 0.20$^a$ | 18.13 ± 4.97$^a$ | 26.62 ± 4.59$^a$ |

Values are mean ± s.e.m. $^a$Means with the same letter are not significantly different.

**Table IV** Cholesterol, lipid and protein content in AH-130 cells

| Treatment | n | Cholesterol Total | Cholesterol Free | Triglycerides | Phospholipids | Proteins |
|---|---|---|---|---|---|---|
| None | 9 | 146 ± 13$^a$ | 56.2 ± 8.4$^a$ | 254 ± 13$^a$ | 184 ± 18$^a$ | 7.38 ± 1.03$^a$ |
| IgGs | 5 | 128 ± 17$^a$ | 43.7 ± 3.4$^a$ | 209 ± 25$^a$ | 182 ± 14$^a$ | 7.40 ± 0.32$^a$ |
| Anti-TNF | 5 | 115 ± 11$^a$ | 59.5 ± 2.2$^a$ | 222 ± 18$^a$ | 199 ± 28$^a$ | 8.37 ± 1.11$^a$ |

Values are mean ± s.e.m. expressed as µg 10$^{-7}$ cells except for proteins (mg 10$^{-7}$ cells). $^a$Means with the same letter are not significantly different.

**Table V** Cholesterol, lipid and protein content in serum of AH-130 tumour-bearing rats

| Animals and treatment | n | Total cholesterol (mg dl$^{-1}$) | Triglycerides (mg dl$^{-1}$) | Phospholipids (mg dl$^{-1}$) | Free fatty acid (µequiv. l$^{-1}$) | Proteins (mg ml$^{-1}$) |
|---|---|---|---|---|---|---|
| Controls | 4 | 85.3 ± 7.9$^a$ | 100 ± 11$^a$ | 63.6 ± 5.1$^a$ | 834 ± 63.7$^a$ | 68.4 ± 2.5$^a$ |
| AH-130 hosts | | | | | | |
| None | 9 | 99.2 ± 3.5$^a$ | 334 ± 38$^b$ | 70.8 ± 5.6$^a$ | 1852 ± 80.2$^b$ | 56.4 ± 2.8$^b$ |
| IgGs | 5 | 112.0 ± 7.1$^a$ | 397 ± 23$^b$ | 79.6 ± 4.5$^a$ | 1984 ± 99.0$^b$ | 57.2 ± 3.8$^b$ |
| Anti-TNF | 5 | 102.8 ± 6.4$^a$ | 213 ± 23$^{b,c}$ | 67.0 ± 6.6$^a$ | 1263 ± 92.8$^{b,c}$ | 69.4 ± 5.2$^c$ |

Values are mean ± s.e.m. $^{a,b,c}$Means with different letters are significantly different ($^bP<0.05$ vs controls, $^cP<0.05$ vs untreated AH-130 hosts).

**Table VI** Cholesterol, lipid and protein composition of HDL lipoproteins in AH-130 tumour-bearing rats

| Animals and treatments | n | Cholesterol (mg dl$^{-1}$) | Triglycerides (mg dl$^{-1}$) | Phospholipids (mg dl$^{-1}$) | Proteins (mg ml$^{-1}$) |
|---|---|---|---|---|---|
| Controls | 4 | 55.8 ± 2.7$^a$ | 3.7 ± 0.7$^a$ | 28.8 ± 5.1$^a$ | 56.4 ± 3.9$^a$ |
| AH-130 hosts | | | | | |
| None | 9 | 39.9 ± 2.7$^b$ | 15.5 ± 1.1$^b$ | 17.6 ± 1.7$^c$ | 40.0 ± 3.8$^c$ |
| IgGs | 5 | 38.8 ± 6.0$^b$ | 19.7 ± 0.7$^b$ | 14.6 ± 2.8$^c$ | 35.9 ± 4.0$^c$ |
| Anti-TNF | 5 | 42.8 ± 4.0$^b$ | 14.4 ± 1.7$^b$ | 15.8 ± 3.0$^c$ | 53.8 ± 5.0$^d$ |

Values are mean ± s.e.m. $^{a,b,c,d}$Means with different letters are significantly different ($^bP<0.01$, $^cP<0.05$ vs controls; $^dP<0.05$ vs untreated AH-130 hosts).

**Table VII** Cholesterol, lipid and protein composition of VLDL-LDL lipoproteins in AH-130 tumour-bearing rats

| Animals and treatments | n | Cholesterol (mg dl$^{-1}$) | Triglycerides (mg dl$^{-1}$) | Phospholipids (mg dl$^{-1}$) | Proteins (mg ml$^{-1}$) |
|---|---|---|---|---|---|
| Controls | 4 | 21.6 ± 1.3$^a$ | 85.2 ± 8.3$^a$ | 10.4 ± 0.9$^a$ | 5.4 ± 0.3$^a$ |
| AH-130 hosts | | | | | |
| None | 9 | 52.7 ± 3.5$^b$ | 247.0 ± 33.8$^b$ | 41.1 ± 3.7$^b$ | 9.5 ± 0.5$^b$ |
| IgGs | 5 | 37.5 ± 2.3$^c$ | 354.0 ± 14.2$^{b,d}$ | 52.5 ± 2.5$^b$ | 11.9 ± 2.2$^b$ |
| Anti-TNF | 5 | 41.0 ± 2.2$^c$ | 177.0 ± 23.3$^{c,d}$ | 50.1 ± 4.4$^b$ | 10.5 ± 0.9$^b$ |

Values are mean ± s.e.m. $^{a,b,c,d}$Means with different letters are significantly different ($^bP<0.01$, $^cP<0.05$ vs controls; $^dP<0.05$ vs untreated AH-130 hosts).

lipogenesis, resulting in increased concentrations of circulating lipids (Feingold and Grunfeld, 1987; Starnes *et al.*, 1988; Evans *et al.*, 1989; Grunfeld *et al.*, 1989). In the adipose tissue this cytokine inhibits the synthesis of LPL (Kawakami *et al.*, 1982) as well as the synthesis of acetyl-CoA carboxylase (Pape and Kim, 1988), fatty acid synthetase (Pekala *et al.*, 1983), fatty acid binding protein and glycerol phosphate dehydrogenase (Torti *et al.*, 1985), all of which are involved in lipid synthesis. TNF also stimulates triglyceride degradation in adipocytes by activating the hormone-sensitive lipase (Pekala *et al.*, 1984).

*In vivo* administation of this cytokine results in decreased LPL activity in the adipose tissue (Semb *et al.*, 1987; Evans and Williamson, 1988). In a recent study by Ettinger *et al.* (1990), total plasma cholesterol was reported to be decreased in monkeys treated with TNF (or LPS), due to a reduction in both LDL and HDL fractions and in association with low lecithin–cholesterol acyltransferase activity; moreover, plasma triglycerides were increased, as commonly observed after TNF administration. By contrast, other authors have observed increased serum cholesterol levels after TNF administration (Feingold and Grunfeld, 1987).

These observations support the conclusion that TNF may be involved in the alterations of lipid metabolism that affect

**Figure 1** Separation by HPLC of serum lipoproteins in AH-130 tumour-bearing rats. Peaks: 1, VLDL; 2, LDL; 3, HDL; 4, $HDL_3$; 5, other serum proteins. (**a**) Controls. (**b**) AH-130 hosts. (**c**) AH-130 hosts + IgGs. (**d**) AH-130 hosts + anti-TNF.

the AH-130 tumour bearers (Carbò *et al.*, 1994). However, treatment with anti-TNF only partially corrected tumour-induced perturbations of lipid metabolism. In particular, triglyceridaemia and free fatty acid levels were reduced, while anti-TNF treatment did not affect total cholesterolaemia and HDL levels.

Cell proliferation, either normal or neoplastic, is commonly associated with altered cholesterol metabolism, and in particular with decreased plasma HDL-cholesterol levels (Dessì *et al.*, 1986, 1989, 1992*b*). It has been proposed that this reduction results from a decrease in the release of cholesterol from proliferating cells to HDL (Daniels *et al.*, 1987). Exogenous cholesterol and cell growth rate modulate the activity of specific HDL receptors; the binding with HDL promotes selective removal of excess cholesterol from the intracellular pool (Oram *et al.*, 1987). It has been shown that both HDL-mediated efflux and HDL receptor activity are down-regulated in actively proliferating cells (Bierman *et al.*, 1989), likely resulting in the reduction of HDL-cholesterol plasma levels.

In the present paper we demonstrate that the anti-TNF treatment is unable to either modify the growth rate of the AH-130 hepatoma or to prevent the decrease in plasma HDL-cholesterol, further supporting the existence of a close relationship between these two parameters.

On the whole, these observations suggest that the hypertriglyceridaemia and the increase of VLDL and LDL, and the increase of HDL metabolism in rats bearing the AH-130 tumour are regulated, at least in part, by different mechanisms. Decreased LPL activity mediated by TNF could well account for the former. By contrast, TNF does not appear to be directly involved in the perturbations of HDL metabolism, which seem to be strictly related to tumour proliferation rates.

**Acknowledgements**

Work supported by the Ministero dell'Università e della Ricerca Scientifica e Tecnologica (40% and 60% funds), Rome, the Consiglio Nazionale delle Ricerche (Special Project ACRO), Rome, the Associazione Italiana per la ricerca sul Cancro, Milan, Regione Autonoma Sardinia, and National Institutes of Health, (grant no. GM32654, Bethesda, MD, Dr Bagby's research on antibodies against TNF).

**References**

ANDERSON RGW, BROWN MS AND GOLDSTEIN JL. (1981). Deficient internalization of receptor-bound low density lipoprotein in human A-431 cells. *J. Cell Biol.*, **88**, 441-452.

BAGBY GJ, PLESSALA KL, WILSON LA, THOMPSON JJ AND NELSON S. (1991). Divergent efficacy of anti-TNF antibody in intravascular and peritonitis model of sepsis. *J. Infect. Dis.*, **163**, 83-88.

BANI IA, WILLIAMS CM, BOULTER PS AND DICKERSON JWT. (1986). Plasma lipids and prolactin in patients with breast cancer. *Br. J. Cancer*, **54**, 439-446.

BEUTLER B AND CERAMI A. (1988). The history, properties, and biological effects of cachectin. *Biochemistry*, **27**, 7575-7582.

BIERMAN EL, OPPENHEIMER M AND ORAM JF. (1989). The regulation of HDL receptor radioactivity. In: *Atherosclerosis VIII*, Crepaldi G, Gotto AM, Manzato E and Baggio G (eds) pp. 297-300. Excerpta Medica: Amsterdam.

BOER GJ. (1975). A simplified microassay of DNA and RNA using ethidium bromide. *Anal. Biochem.*, **193**, 225-231.

BOWMAN RE AND WOLF, RC. (1962). A rapid and specific ultramicromethod for total serum cholesterol. *Clin. Chem.*, **8**, 302-309.

CARBÒ N, COSTELLO P, TERRITORE L, BAGBY GJ, LOPES-SORIANO FJ, BACCINO FM AND ARGILES JM. (1994). Anti-tumour necrosis factor-α treatment interferes with changes in lipid metabolism in a tumour cachexia model. *Clin. Sci.*, **87**, 349-355.

CLARK RW AND CRAIN RC. (1986). Characterization of alterations in plasma lipoprotein lipid and apoprotein profiles accompanying hepatoma-induced hyperlipidemia in rats. *Cancer Res.*, **46**, 1894-1903.

COLEMAN PS AND LAVIETES BB. (1981). Membrane cholesterol tumorigenesis and the biochemical phenotype of neoplasia. *CRC Crit. Rev. Biochem.*, **11**, 341-393.

COSTELLI P, CARBÒ N, TESSITORE L, BAGBY GJ, LOPEZ-SORIANO FJ, ARGILES JM AND BACCINO FM. (1993). Tumor necrosis factor-α mediates changes in tissue protein turnover in a rat cancer cachexia model. *J. Clin. Invest.*, **92**, 2783-2789.

DANIELS RJ, GUERTLER LS, PARCHER TS AND STEINBERG D. (1987). Studies on the rate of efflux of cholesterol from cultured human skin fibroblasts. *J. Biol. Chem.*, **256**, 4978-4983.

DESSÌ S, CHIODINO C, BATETTA B, FADDA AM, ANCHISI C AND PANI P. (1986). Hepatic glucose-6-phosphate dehydrogenase, cholesterogenesis and serum lipoproteins in liver regeneration after partial hepatectomy. *Exp. Mol. Pathol.*, **44**, 169-176.

DESSÌ S, BATETTA B, CARRUCCIU A, PULISCI D, LACONI S, FADDA AM, ANCHISI C AND PANI P. (1989). Variation of serum lipoproteins during cell proliferation induced by lead nitrate. *Exp. Mol. Pathol.*, **51**, 97-102.

DESSÌ S, BATETTA A, PULISCI D, ACCOGLI P, PANI P AND BROCCIA G. (1991). Total and HDL cholesterol in human hematologic neoplasms. *Int. J. Hematol.*, **54**, 483-486.

DESSÌ S, BATETTA B, ANCHISI C, PANI P, COSTELLI P, TESSITORE L AND BACCINO FM. (1992a). Cholesterol metabolism during the growth of a rat ascites hepatoma (Yoshida AH-130). *Br. J. Cancer*, **66**, 787-793.

DESSÌ S, BATETTA B, PULISCI D, SPANO O, CHERCHI R, LANFRANCO G, TESSITORE L, COSTELLI P, BACCINO FM AND PANI P. (1992a). Altered pattern of lipid metabolism in patients with lung cancer. *Oncology*, **49**, 436-441.

DESSÌ S, BATETTA B, PULISCI D, SPANO O, ANCHISI C, TESSITORE L, COSTELLI P, BACCINO FM, AROASIO E AND PANI P. (1994). Cholesterol content in tumor tissues is inversely associated with high-density lipoprotein cholesterol in serum in patients with gastrointestinal cancer. *Cancer*, **73**, 253-258.

DIETSCHY JN, TURLEY SD AND SPADY DK. (1993). Role of liver in the maintenance of cholesterol and low density lipoprotein homeostasis in different animals species including humans. *J. Lipid Res.*, **34**, 1637-1659.

EISENBERG S. (1984). High desnity lipoprotein metabolism. *J. Lipid Res.*, **25**, 1017-1058.

ETTINGER WH, MILLER LD, ALBERTS JJ, SMITH TK AND PARKS JS. (1990). Lipopolysaccharide and tumor necrosis factor cause a fall in plasma concentration of lecithin cholesterol acyltransferase in cynomolgus monkey. *J. Lipid Res.*, **31**, 1099-1107.

EVANS RD AND WILLIAMS DH. (1988). Tumour necrosis factor-α (cachectin) mimics some of the effects of tumour growth on the disposal of a [$^{14}$C]lipid load in virgin, lactating and litter-removed rats. *Biochem. J.*, **256**, 1055-1058.

EVANS RD, ARGILES JM AND WILLIAMSON DH. (1989). Metabolic effects of tumour necrosis factor-α (cachectin) and interleukin-1. *Clin. Sci.*, **77**, 357-364.

FEINGOLD KR AND GRUNFELD C. (1987). Tumor necrosis factor alpha stimulates hepatic lipogenesis in the rat in vivo. *J. Clin. Invest.*, **80**, 184-190.

FEINGOLD KR, STAPRANS I, MEMON RA, MOSER AH, SHIGENAGA, JK, DOERRIER W, DINARELLO CA AND GRUNFELD C. (1992). Endotoxin rapidly induces changes in lipid metabolism that produce hypertriglyceridemia: low doses stimulate hepatic triglyceride production while high doses inhibit clearance. *J. Lipid Res.*, **33**, 1765-1776.

FEINGOLD KR, HARDARDOTTIR I, MEMON R, KRUL EJT, MOSER AH, TAYLOR JM AND GRUNFELD C. (1993). Effect of endotoxin on cholesterol biosynthesis and distribution in serum lipoproteins in symian hamsters. *J. Lipid Res.*, **34**, 2147-2158.

FOLCH J, LEES, M AND SLOANE-STANLEY GH. (1957). A simple method for the isolation and purification of total lipids from animals tissues. *J. Biol. Chem.*, **226**, 497-509.

GRUNFELD C, WILKING H, NEESE R, GAVIN RA, MOSER AH, GULLI R, KERALA-SERIO M AND FEINGOLD KR. (1989). Persistence of hypertriglyceridemic effect of tumor necrosis factor despite development of tachyphylaxis to its anorectic/cachectic effects in rats. *Cancer Res.*, **49**, 2554-2560.

HARADA K, SHIMANO H, KAWAKAMI M, ISHIBASHI S, GOTODA T, MORI N, TAKAKU F AND YAMADA N. (1990). Effect of tumor necrosis factor/cachectin on the activity of the low density lipoprotein receptor on human skin fibroblasts. *Biochem. Biophys. Res. Comm.*, **172**, 1022-1027.

HAVEL RJ, EDER HA AND BRAGDEN JH. (1955). The distribution and chemical composition of ultracentrifugally separated lipoproteins in human serum. *J. Clin. Invest.*, **34**, 1345-1353.

KANNAN R AND BAKER N. (1977). Hypertriglyceridemia in Ehrlich ascites carcinomatous mice: tumor and mouse strain differences. *Lipids*, **12**, 153-158.

KAWAKAMI M, PEKALA PH, LANE MD AND CERAMI A. (1982). Lipoprotein lipase suppression in 3T3-Li cells by an endotoxin-induced mediator from exudate cells. *Proc. Natl Acad. Sci. USA*, **79**, 912-916.

LOWRY OH, ROSEBROUGH NJ, FARR AL AND RANDALL RJ. (1951). Protein measurement with the Folin phenol reagent. *J. Biol. Chem.*, **193**, 265-275.

OKAZAKY M, OHNO Y AND HARA I. (1980). High performance aqueous gel permeation chromatography of human serum lipoproteins. *J. Chromatogr.*, **21**, 257-264.

ORAM JF, JOHNSON C AND BROWN TA. (1987). Interaction of high density lipoprotein with its receptor on cultured fibroblasts and macrophages. *J. Biol Chem.*, **262**, 2405-2410.

PAPE PE AND KIM KH. (1988). Effect of tumor necrosis factor on acetyl-coenzyme A carboxylase gene expression and preadipocyte differentiation. *Mol. Endocrinol.*, **2**, 395-403.

PEKALA PH, KAWAKAMI M, ANGUS CW, LANE MD AND CERAMI A. (1983). Selective inhibition of synthesis of enzymes for the novo fatty acid biosynthesis by an endotoxin-induced mediator from exudate cells. *Proc. Natl Acad. Sci. USA*, **80**, 2743-2747.

PEKALA PH, PRICE SR, HORN CA, HOM BE, MOSS J AND CERAMI A. (1984). Model for cachexia in chronic disease: secretory products of endotoxin-stimulated macrophages induce a catabolic state in 3T3-L1 adipocytes. *Trans. Assoc. Am. Physicians.*, **97**, 251-259.

REVERTER JC, SIERRA J, MARTI-TULUSAUS JM, MONSERRAT E, GRANENA A AND ROZMAN C. (1988). Hypocholesterolemia in acute myelogenous leukemia. *Eur. J. Haematol.*, **41**, 317-320.

ROSSNER S AND WALLGREN A. (1984). Serum lipoproteins and proteins after breast cancer surgery and effects of tamoxifen. *Atherosclerosis*, **52**, 339-346.

SEMB H, PETERSON J, TAVERNIER J AND OLIVECRONA T. (1987). Multiple effects of tumor necrosis factor on lipoprotein lipase in vivo. *J. Biol. Chem.*, **262**, 8390-8394.

STARNES HF, WARREN RS, JEEVANANDAM M, GABRILOVE JL, LARKIAN W, OETTGEN HF AND BRENNAN MF. (1988). Tumor necrosis factor and the acute metabolic response to tissue injury in man. *J. Clin. Invest.*, **82**, 1321-1325.

TESSITORE L, BONELLI G AND BACCINO FM. (1987). Early development of protein metabolic perturbations in the liver and skeletal muscle of tumour-bearing rats. *Biochem. J.*, **241**, 153-159.

TESSITORE L, COSTELLI P AND BACCINO FM. (1993). Humoral mediation for cachexia in tumour-bearing rats. *Br. J. Cancer*, **67**, 15-23.

TORTI FM, DIECKMAN B, BEUTLER B, CERAMI A AND RINGOLD GM. (1985). A macrophage factor inhibits adipocytes gene expression: an in vitro model of cachexia. *Science*, **229**, 867-869.

VAN HANDEL E AND ZILVERSMIT DB. (1968). Micromethod for the direct determinations of serum triglycerides. *J. Lab. Clin. Med.*, **50**, 152-157.

VITOLS S, GAHRTON G, BJORKHOLM M AND PETERSON C. (1985). Hypocholesterolaemia in malignancy due to elevated low density lipoproteins receptor activity in tumour cell: evidence from studies in patients with leukaemia. *Lancet*, **2**, 1150-1154.

YACHNIN S, LARSON A AND WEST EJ. (1983). Rates of cholesterol biosynthesis are related to early differentiation in acute nonlymphocytic leukaemia cells. *Br. J. Haematol.*, **54**, 459-466.